# Deep Multi-OMICs and Multi-Tissue Characterization in a Pre- and Postprandial State in Human Volunteers: The GEMM Family Study Research Design

**DOI:** 10.3390/genes9110532

**Published:** 2018-11-02

**Authors:** Raul A. Bastarrachea, Hugo A. Laviada-Molina, Edna J. Nava-Gonzalez, Irene Leal-Berumen, Claudia Escudero-Lourdes, Fabiola Escalante-Araiza, Vanessa-Giselle Peschard, Rosa A. Veloz-Garza, Karin Haack, Angélica Martínez-Hernández, Francisco M. Barajas-Olmos, Fernanda Molina-Segui, Fatima A. Buenfil-Rello, Lucia Gonzalez-Ramirez, Reinhard Janssen-Aguilar, Ricardo Lopez-Muñoz, Fernanda Perez-Cetina, Janeth F. Gaytan-Saucedo, Zoila Vaquera, Judith Cornejo-Barrera, Juan Carlos Castillo-Pineda, Areli Murillo-Ramirez, Sara P. Diaz-Tena, Benigno Figueroa-Nuñez, Laura González-López, Rocío A. Salinas-Osornio, Melesio E. Valencia-Rendón, José Ángeles-Chimal, Jesús Santa-Olalla Tapia, José M. Remes-Troche, Salvador B. Valdovinos-Chavez, Eira E. Huerta-Avila, Xianlin Han, Lorena Orozco, Ernesto Rodriguez-Ayala, Susan Weintraub, Esther C. Gallegos-Cabrales, Shelley A. Cole, Jack W. Kent

**Affiliations:** 1 Department of Genetics, Texas Biomedical Research Institute, San Antonio, TX 78227-0549, USA; khaack@txbiomed.org (K.H.); maryferpcetina@hotmail.com (F.P.-C.); janethgaytan@gmail.com (J.F.G.-S.); ZVaquera@txbiomed.org (Z.V.); lneeha920609@gmail.com (E.E.H.-A.); scole@txbiomed.org (S.A.C.); jkent@txbiomed.org (J.W.K.J.); 2 Escuela de Ciencias de la Salud, Universidad Marista de Mérida, Mérida 97300, Mexico; hlaviada@marista.edu.mx (H.A.L.-M.); fmolina@marista.edu.mx (F.M.-S.); ln.fatimabuenfil@hotmail.com (F.A.B.-R.); nut.lucia.gonzalez@hotmail.com (L.G.-R.); rjanssen91@gmail.com (R.J.-A.); Lopezm.ric@gmail.com (R.L.-M.); 3 Facultad de Salud Pública y Nutrición (FASPyN), UANL, Monterrey 64460, Mexico; edna.navag@uanl.mx; 4 Facultad de Medicina y Ciencias Biomédicas, Universidad Autónoma de Chihuahua, Chihuahua 31125, Mexico; ileal@uach.mx; 5 Facultad de Ciencias Químicas, Universidad Autónoma de San Luis Potosí, San Luis Potosí 78240, Mexico; cescuder@uaslp.mx; 6 Centro de Investigación en Ciencias de la Salud (CICSA), Facultad de Ciencias de la Salud, Universidad Anáhuac Norte, Lomas Anahuac 52786, Mexico; fescara@gmail.com (F.E.-A.); vanessa-gisellep@wustl.edu (V.-G.P.); ernesto.rodriguez@anahuac.mx (E.R.-A.); 7 Facultad de Enfermería, Universidad Autónoma de Nuevo León (UANL), Monterrey 64460, Mexico; rosyvelozgarza@hotmail.com (R.A.V.-G.); svmonterrey@gmail.com (S.B.V.-C.); esther.gallegosc@gmail.com (E.C.G.-C.); 8 Laboratorio de Inmunogenómica y Enfermedades Metabólicas, Instituto Nacional de Medicina Genómica, Ciudad de México C.P. 14610, Mexico; amartinez@inmegen.gob.mx (A.M.-H.); fbarajas@inmegen.gob.mx (F.M.B.-O.); lorozco@inmegen.gob.mx (L.O.); 9 Departamento de Enseñanza, Postgrado e Investigación, Hospital Infantil de Tamaulipas, Ciudad Victoria 87150, Mexico; judith.cornejo@tam.gob.mx; 10 Departamento de Nutrición Humana, Universidad Latina de América, Morelia, Michoacán 58170, Mexico; castillomorelia@hotmail.com (J.C.C.-P.); amurillor@unla.edu.mx (A.M.-R.); ln.sarapatriciadiaz@victoriamedicalcenter.com (S.P.D.-T.); 11 Clínica de Enfermedades Crónicas y Procedimientos Especiales (CECYPE), Morelia 58249, Mexico; benigno.figueroa@cecype.com; 12 Universidad del Valle de Atemajac (UNIVA), Zapopan, Jalisco 45050, Mexico; laura.gonzalez@univa.mx (L.G.-L.); rocio.salinas@univa.mx (R.A.S.-O.); tantarrea@gmail.com (M.E.V.-R.); 13 Facultad de Medicina, Universidad Autónoma Estado de Morelos, Cuernavaca 62209, Mexico; chimal@uaem.mx (J.Á.-C.); jsa@uaem.mx (J.S.-O.T.); 14 Instituto de Investigaciones Médico-Biológicas, Universidad Veracruzana, Veracruz 91700, Mexico; jose.remes.troche@gmail.com; 15 Department of Medicine, Sam and Ann Barshop Institute for Longevity and Aging Studies, University of Texas Health San Antonio, San Antonio, TX 78229, USA; HanX@uthscsa.edu; 16 Department of Biochemistry, University of Texas Health Science Center, San Antonio, TX 78229, USA; weintraub@uthscsa.edu

**Keywords:** multi-OMICS, GEMM family study, postprandial metabolism, mixed meal challenge

## Abstract

Cardiovascular disease (CVD) and type 2 diabetes (T2D) are increasing worldwide. This is mainly due to an unhealthy nutrition, implying that variation in CVD risk may be due to variation in the capacity to manage a nutritional load. We examined the genomic basis of postprandial metabolism. Our main purpose was to introduce the GEMM Family Study (Genetics of Metabolic Diseases in Mexico) as a multi-center study carrying out an ongoing recruitment of healthy urban adults. Each participant received a mixed meal challenge and provided a 5-hours’ time course series of blood, buffy coat specimens for DNA isolation, and adipose tissue (ADT)/skeletal muscle (SKM) biopsies at fasting and 3 h after the meal. A comprehensive profiling, including metabolomic signatures in blood and transcriptomic and proteomic profiling in SKM and ADT, was performed to describe tendencies for variation in postprandial response. Our data generation methods showed preliminary trends indicating that by characterizing the dynamic properties of biomarkers with metabolic activity and analyzing multi-OMICS data it could be possible, with this methodology and research design, to identify early trends for molecular biology systems and genes involved in the fasted and fed states.

## 1. Introduction

Mexicans share with Mexican Americans an elevated risk of cardiovascular diseases (CVD) and type 2 diabetes (T2D) [1]. In the US, Mexican Americans have the highest age-adjusted prevalence of the metabolic syndrome (31.9%) [2]. In a population-based survey in the Republic of Mexico, the prevalence of the metabolic syndrome was 26.6% [3]. This shared, elevated prevalence of CVD risk factors suggests shared genetic factors. As the source population, Mexico reflects the allelic diversity resulting from the conquest and subsequent confluence of European and Native American origins, and therefore reflects the full extent of the spectrum of risk [4]. Because Hispanics, including Mexican Americans, are among the fastest growing population groups in the US, knowledge gained from the source population will directly inform public health initiatives in the US [5].

**Figure 1:**
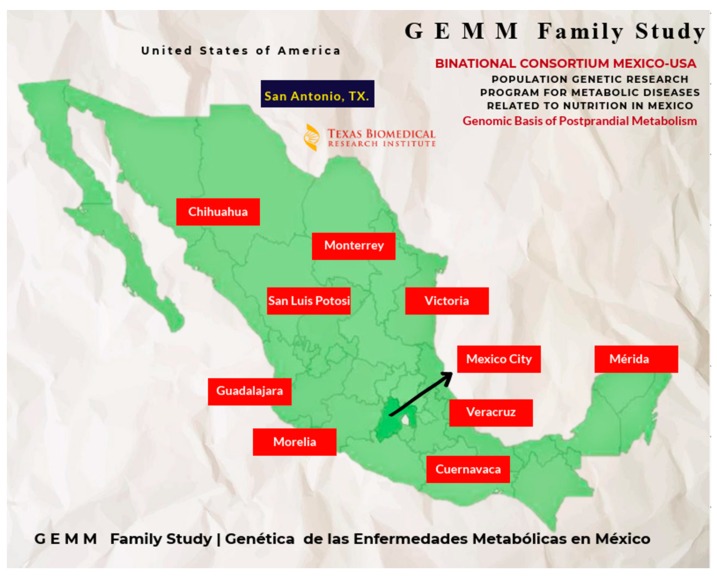
Map of Mexico showing location of GEMM (Genetics of Metabolic Diseases in Mexico) Centers.

The GEMM (Genética de las Enfermedades Metabólicas en México/Genetics of Metabolic Diseases in Mexico Family Study) is a bi-national, multi-center collaborative study of cardiovascular risk phenotypes of metabolic origin (CVRMO) related to T2D and the risk of CVD [6]. The overall goal of this project is to identify the genetic and molecular processes involved in the development of these major public health threats in an effort to better diagnose and treat those who are afflicted or at risk [7]. Scientific oversight and coordination of GEMM is provided by a steering committee comprising investigators at Texas Biomedical Research Institute (Texas Biomed), San Antonio, TX, USA and the Mexican National Institute of Genomic Medicine (INMEGEN). Ten participating centers in Mexico have been carefully selected based on their affiliation with a medical university and/or teaching hospital. Each center has obtained funding in Mexico, including very substantial institutional commitments of resources and personnel, to set up a state-of-the-art diagnostic facility dedicated to GEMM and recruit participants [8] (Figure 1).

The vast majority of studies and clinical diagnosis of metabolic diseases have been focused on the fasted state [9]. However, it has been suggested that atherosclerotic changes start to develop in the pre-diabetic state [10]. Accumulating evidence suggests that postprandial hyperglycemia and elevated levels of postprandial lipoproteins predict higher CVD risk [11]. Postprandial hypertriglyceridemia is a recognized independent predictor of cardiovascular pathology [12]. Our interest is in the normal range of variation of CVRMO phenotypes in apparently healthy individuals, including subtle differences in metabolic processes that can point to novel biomarkers of incipient disease. Deep phenotyping [13], as it is proposed in this study, is likely to improve accuracy in classification of disease outcomes, relative to earlier epidemiological and clinical studies, by providing comprehensive, individualized profiles of risk. 

The GEMM family study design characterizes detailed dynamic and function-based metabolic phenotypes in fasting and fed states (including the phenome, transcriptome, proteome and metabolome) [14]. Data are acquired from the circulation, adipose tissue and skeletal muscle, tissues that are key for understanding insulin action and carbohydrate and lipid homeostasis. All measurements in blood are taken over a time course of 5 h to allow fine-scale profiling of individual postprandial response.

The aims of this paper are (a) to introduce the GEMM family study research design aimed at characterizing the individual response to a mixed meal challenge, in order to define the range of efficiency of nutrient utilization in nominally healthy individuals in a population at risk of cardiometabolic disease; and (b) to present preliminary application of the research design to 16 female participants to show the full clinical and molecular phenotypic characterization expected to occur in our final database.

## 2. Materials and Methods

### 2.1. Recruitment of Study Participants

**Figure 2:**
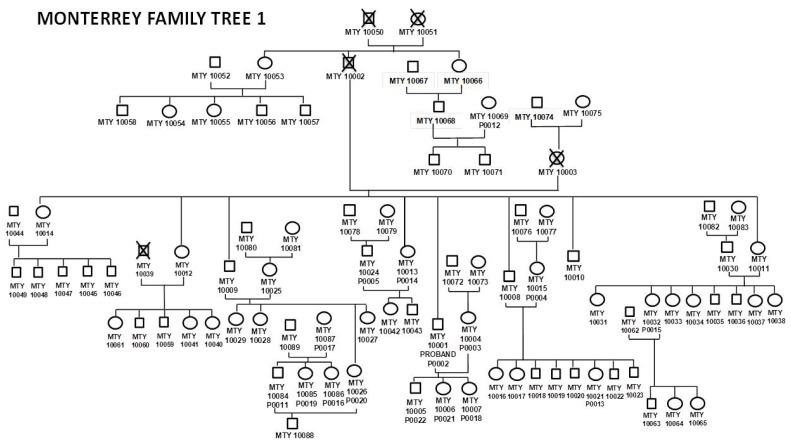
Three-generation family tree from Monterrey, Mexico as an example of the typical extended pedigrees recruited in the GEMM Study. Proband: MTY1001 (P002). Proband’s wife: MTY1002 (P004). Founders: MTY 10050&51 (deceased). Proband’s parents: MTY1002&03 (deceased). Brothers and sisters: MTY 1011, 10, 08, 13 (P0014), 09, 12, 14. Daughters: MTY1006 (P021) and 07 (P018). Son: MTY 1005 (P022). P00: Participants that have already volunteered for the postprandial/biopsy procedures. Female—circles; Male—squares.

Families are ascertained and recruited following established guidelines and strategies for processes of recruitment in prospective family-based studies [15]. The ideal proband for recruitment is healthy, aged 25–45 years, and both parents are also healthy, alive and willing to participate. Once a proband is enrolled, an invitation to participate in the study will be extended to all relatives of first, second or third degree (e.g., parents, children, grandparent/grandchild, avuncular, cousins, etc.) who are at least 18 years of age, their spouses, and the proband’s spouse and the spouse’s relatives aged 18 or above. Our goal is to recruit 400 healthy volunteers in ~10 extended families (40 subjects from each of the participating centers), ascertained without regard to disease status [16]. Figure 2 represents the pedigree of a typical family recruited in this study from Monterrey, Nuevo Leon, Mexico. All participants provide written, informed consent, and all procedures are approved by the Ethical Committees (Institutional Review Boards) of the respective centers. Export of GEMM samples for multi-OMICs analysis to the USA. has been permitted by the Mexican Federal government in accordance with Mexican genetic sovereignty law [17] (COFEPRIS Permit No. COF187278 (DEAPE 133300CT190038/2013) issued on 19 March 2013) [18]. This family-based study is currently approved by the Institutional Review Board at the University of Texas Health Sciences Center at San Antonio (IRB Number HSC20170448H) and is conducted according to the principles expressed in the declaration of Helsinki. 

### 2.2. Study Timeline and Standardization of Postprandial Procedures

The capability for each center is up to four individuals a month when the Center is in operation. A typical day in the center is as follows: 

#### 2.2.1. First Visit

Study participant arrives after a 12 h overnight fast. On arrival, fasting anthropometric measurements were recorded: weight, height, waist circumference, body mass index (BMI), and body composition by bioimpedance. Blood pressure and heart rate is also measured. First (fasting) measurement of energy expenditure with MedGem (Microlife, Clearwater Florida, USA), an indirect calorimetry clinically-validated and Food and Drug Administration (FDA)-approved medical device for measurement of resting metabolic rate (RMR) [19]. An IV line and catheter are placed in the median basilic vein of the forearm and a fasting blood sample (#0, time 0 min) is taken. Immediately after, participant ingests his/her mixed meal as 30% of Total Daily Energy Expenditure (TDEE). Postprandial blood samples #1 (15 min), #2 (30 min), #3 (45 min), #4 (60 min), #5 (90 min) follow. Second evaluation of RMR with MedGem (postprandial) is performed. Postprandial sample #6 (120 min) and #7 (180 min) follow. Immediately after, postprandial muscle (100 mg) and subcutaneous adipose tissue (160 mg) biopsies are obtained from the right thigh by a surgeon, serving as the postprandial tissue biopsy 3 h after meal ingestion. Postprandial blood samples #8 (240 min) and #9 (300 min) are taken. Third evaluation of RMR with MedGem (postprandial) is performed. Immediately after, the IV catheter is removed, subject is given lunch and dismissed. 

#### 2.2.2. Second Visit (14 Days after the First Visit)

On arrival after a 12 h fast, total body bone and tissue composition densitometry (DXA-GE Lunar Prodigy GE Healthcare, Madison, WI, USA) is performed. Blood pressure and heart rate are measured. Immediately after, the second muscle (100 mg) and adipose tissue (160 mg) biopsies in fasting are obtained from the left thigh. Subject is given breakfast and dismissed. 

### 2.3. Anthropometric Measurement, Body Composition and Indirect Calorimetry

Waist circumference is measured with a professional Gulick tape measure (North Coast Medical, Inc., Morgan Hill, CA, USA) in centimeters. Height is determined by a fixed HM200P Portstad Portable stadiometer (Quick Medical, Issaquah, WA, USA). Methods used for measurement of weight and bioimpedance (Tanita BC—418 Body Composition Analyzer, (Hanover, MD, USA) [20], body composition by dual energy X-ray absorptiometry (DXA-GE Lunar Prodigy) [21]), and RMR by indirect calorimetry (MedGem) have been described previously [19]. 

### 2.4. Mixed Meal Challenge

Having fasted overnight, each participant consumes a defined mixed meal (Ensure Plus^®^; Abbott Nutrition, Lake Forest, IL, USA) containing 57% of total calories from carbohydrates, 28% from fat, and 15% from protein. The amount of the mixed meal provided is calculated by a staff dietician to provide 30% of the participant’s daily energy requirement based on total body weight (Harris–Benedict equation) and fat free mass (kg) (Katch–McArdle formula) [22]. 

### 2.5. Hormone, Cytokine and Clinical Chemistry Measurements for Postprandial Metabolic Assessments

Biochemical phenotypes are analyzed on a Luminex 100IS platform (SBH Sciences, Natick, MA, USA) and an Immulite 1000 (Diamond Diagnostics Inc., Holliston, MA, USA) which run enzyme-linked immunosorbent assay (ELISA) and radioimmunoassay (RIA) analyses. We measure a wide range of clinical biochemistries, hormones, cytokines and endophenotypes (Table 1, Table 2 and Table 3). These analyte categories included: Gastrointestinal biomarkers (satiety signals: GLP-1, PYY, Ghrelin; β cell and insulin-glucose axis: insulin, glucose, HOMA-IR, Matsuda Index; Adipose tissue function: leptin, adiponectin; Inflammation endophenotypes: C-RP high sensitive, PAI-1, TNF-α, MCP-1; Lipid-lipoprotein metabolism: NEFA, tryglycerides, HDL-C.)

### 2.6. Fasting and Postprandial Plasma Metabolomic Profiling of Amino Acids and Acylcarnitines

The derivatized amino acids are analyzed by high-performance liquid chromatography-electrospray ionisation–mass spectrometry (HPLC-ESI-MS) on a Q Exactive mass spectrometer (Thermo Fisher Scientific, Austin, TX, USA) used together with a Dionex Ultimate 3000 HPLC (Thermo Fisher Scientific) [23]. Extracted ion chromatograms were generated for the protonated molecule for each derivatized amino acid using a mass window of ±5 ppm. Peak areas are determined by processing through TraceFinder (Thermo Fisher Scientific) and compared to calibration curves generated by analysis of authentic standards. 

### 2.7. Acylcarnitines

Lipids are extracted using ice-cold chloroform/methanol (2:1) (Millipore Sigma, St. Louis, MO, USA), with addition of water as needed to generate a two-phase system. A bead homogenizer was used for tissue homogenization. Palmitoyl-[1,2,3,4-^13^C_4_] L-carnitine internal standard (Adventbio Chyrstal Chem, Elk Grove Village, IL, USA) is added at the time of extraction. After centrifugation the chloroform layer was removed, dried in vacuo and reconstituted in injection solution. HPLC-ESI-tandem-MS analyses was conducted on a Q Exactive mass spectrometer (Thermo Fisher) used in conjunction with a Thermo Fisher/Dionex Ultimate 3000 HPLC (Thermo Fisher Scientific) [24]. TraceFinder (Thermo Fisher Scientific) was used for processing of the quantitative data.

### 2.8. Transcriptomics: RNA Gene Expression Profiling

Total RNA is isolated from skeletal muscle and subcutaneous adipose tissue [25]. Messenger RNA (mRNA) (from total RNA) is converted into a complementary DNA (cDNA) library using the Illumina TruSeq Stranded mRNA sample preparation kit (Illumina Inc., San Diego, CA, USA). PolyA mRNAs is preferentially selected from 0.1–4 ug of high-quality total RNA using magnetic beads covered with poly-T oligos (TriLink Biotechnologies, San Diego, CA, USA). The mRNAs undergo a first and second strand cDNA synthesis resulting in a double-strand complementary DNA (dscDNA) with blunt ends. After ligation of an adapter, the products are purified and enriched by polymerase chain reaction (PCR) to create the final cDNA libraries [26]. The preliminary data reported here were generated using Illumina Sentrix Human Whole Genome WG-6 microarrays. We have begun re-analysis of these samples by RNA sequencing and will employ this technology for all future analyses.

### 2.9. Statistical Analysis

For this initial presentation of GEMM data, we obtained summary statistics in R (www.r-project.org) and tests for differences by BMI and feeding status (pre- vs. postprandial). The latter tests were performed using SOLAR [27] to account for the random effect of kinship and included age as covariate. 

### 2.10. Interpretation and Power

The goal of this analysis is to identify those features that are differentially altered by the meal challenge according to prior assessment of cardiometabolic risk, as these are the gene products (and by extension, the genes) most relevant to individual variation in cardiovascular risk of metabolic origin (CVRMO). Given our preliminary data, we expect to find relatively robust effects of both meal challenge and CVRMO stratum. The two-way ANOVA for differential response to meal by stratum will provide 80% power to detect a medium effect of about 6% of variance, based on analysis of ~100 significantly differing features (*p_crit_* = 0.0005). 

### 2.11. Correction for Admixture

The Mexican population has a complex genetic history, blending Native American, European, and Afro-Caribbean ancestry. Our decision to recruit extended families, and to account for explicit relatedness as described in the preceding section, provides one level of adjustment for this non-independence. In addition, we will address potential cryptic relatedness due to population history by performing principal components analysis (PCA) on single nucleotide polymorphism (SNP) data from the Multi-Ethnic AMR/AFR-8 arrays [28] and using principal components as covariates in our analyses to correct for population stratification [29].

## 3. Results

In this section we report baseline and postprandial data from 16 healthy female adult individuals characterized with all fasting and postprandial anthropometric, biochemical, and OMICS data as will be obtained for the full database we are assembling. These data should be appraised as an example of the types of data that the GEMM protocol will collect as well as an assessment of the range of variation in these data. While some of our results are suggestive of possible pathways and mechanisms of metabolic flexibility, we acknowledge that this preliminary sample is too small to be conclusive. Here we present OMICS data from skeletal muscle and plasma from 16 female subjects. Preliminary data for amino acids and acylcarnitines (AcC) only shows branched-chain amino acid (BCCA) profiling and long-chain AcC species (Table 3). Of note, our ongoing protocol has already recruited and collected anthropometric, body composition, buffy coats, fasting and postprandial plasma, subcutaneous adipose and muscle tissue biopsies from 126 healthy volunteers.

These 16 females were divided in two groups by the lower mean (*n* = 8, 24 ± 1.6, max = 26.6, min = 20.7) and higher mean (*n* = 8, 32.1 ± 5.7, max = 41.6, min = 26.8) levels of BMI. Their mean age was 34.1 ± 14.4 y.o. in the lower mean BMI (l.BMI), and 41.2 ± 11.0 y.o. in the higher mean BMI group (h.BMI). Table 1, Table 2 and Table 3 describe their differential fasting and fed physiologic and endophenotypic parameters fully characterized with clinical biochemistries, hormones, cytokines and metabolite measures (amino acids and AcC).

Consistent with other reports, our data showed that leptin levels were higher in the group of h.BMI in the fasting and fed state. However, the fasting circulating levels of leptin in both groups did not change after the mixed meal administration (fasting levels of 8.6 ± 5.6 and postprandial levels (300 min) of 9.4 ± 5.9 ng/mL in the l.BMI group vs. fasting levels of 18.6 ± 9.3 and postprandial levels (300 min) of 16.4 ± 8.2 ng/mL in the h.BMI group, Table 1) [30]. Our methodology proposal/development data also showed fasting adiponectin levels of 19.9 ± 13.2 µg/mL in the l.BMI group and 22.6 ± 19.6 µg/mL in the h.BMI group [31]. 

Among the gut-derived satiety hormones implicated in control of food intake, the orexigenic ghrelin showed higher fasting levels in the subjects with l.BMI (333.5 ± 59.1 pg/mL) compared to subjects with h.BMI (233.1 ± 137.2 pg/mL). The ghrelin pattern followed the documented preprandial rise [32], falling to lower levels within the following 30– 120 min after the mixed meal, rising back again during the next 180 min and 300 min. The pre- and postprandial curves of the satiety peptides GLP-1 and PYY increased after the meal ingestion, returning to fasting levels after 300 min. The fasting pro-inflammatory cytokines measured trended higher in the h.BMI compared to the l.BMI subjects (TNFα: 1.8 ± 0.7 vs. 1.4 ± 1.1 pg/mL; hs-CRP: 0.42 ± 0.30 vs. 0.09 ± 0.07 mg/L; PAI-1: 66,901.4 ± 56,005.3 vs. 43,527.9 ± 38,563.4 pg/mL; IL-6: 2.1 ± 1.6 vs. 1.9 ± 1.3 pg/mL) (Table 1). 

Preprandial and postprandial glucose, insulin, non-esterified free fatty acids (NEFA), tryglcerides and high-density lipoprotein cholesterol (HDL-C) concentrations after the ingestion of the mixed meal challenge are shown in Table 2. Fasting insulin was higher in the in the h.BMI compared to the l.BMI subjects. Postprandial NEFA at 180 min, fasting and postprandial HDL-C at 180 min and 300 min were higher in the in the h.BMI compared to the l.BMI subjects. The homeostasis model assessment (HOMA) index [33] was higher in subjects with h.BMI than in l.BMI participants. Whole-body physiological insulin sensitivity was estimated by calculating the Matsuda index [34]. h.BMI subjects reported an index of 4.89 ± 3.59 compared to l.BMI counterparts reporting 6.66 ± 4.05 (Table 2).

The postprandial quantitative data on amino acids obtained from these 16 healthy females revealed an interesting pattern showing that in the women with the lower BMI the vast majority of essential amino acids was differentially elevated in the fed state (alanine, arginine, asparagine, glutamine, glycine, histidine, isoleucine, leucine, lysine, methionine, proline, serine, threonine, tryptophan, valine) when compared to their counterparts with a higher BMI (aspartic acid, cysteine, glutamic acid, phenylalanine) (Table 3). We also measured AcC in the serum by MS/MS. Among all AcC measured, we focus on the long-chain AcC species (C14:0, C14:1, C14:2, C14:3, C16:0, C16:1, C16:2, C18:0, C18:1, C18:2, C18:3) due to their association with insulin resistance [35]. We observed that only 2 AcC species (30′postprandial AcC C14:3, and 300′ postprandial AcC C16:0), showed a noticeable differential measurement in the h.BMI compared to the l.BMI subjects (Table 3). 

**Figure 3:**
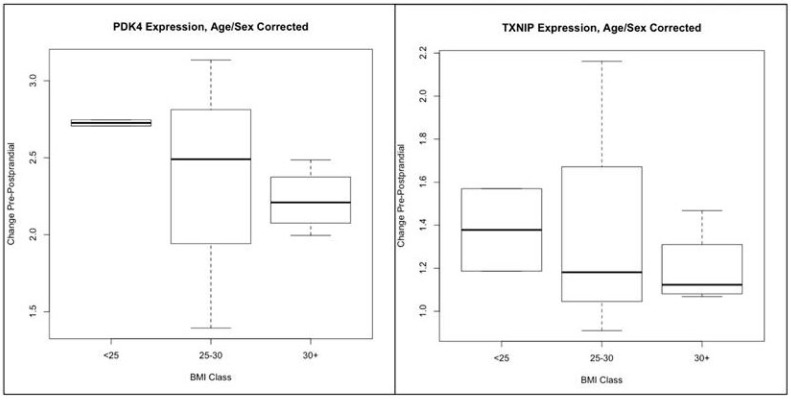
Transcriptomic response to meal in nine GEMM subjects. 32 transcripts in muscle (nominal *p* < 0.05). 15 upregulated, 7 downregulated. Include *PDK4* and *TXNIP* at *p* < 1 × 10^−5^. BMI: body mass index.

For transcriptomics preliminary data we are including a small pilot study of muscle gene expression using transcriptomic array data. Figure 3 and Table 4 show preliminary data for 9 GEMM participants from the same cohort of 16 healthy females for fasting vs. 3 h postprandial skeletal muscle tissue gene expression. These 9 females were divided in three groups (*n* = 3 each) with a BMI < 25, 25–30, and >30–32 transcripts were differentially expressed after the meal at a nominal *p* < 0.05. The two most strongly differentially expressed transcripts in muscle show a similar pattern: lower response in obese vs. healthy weight, and greater variation in the overweight (Figure 3). 

## 4. Discussion

Our discussion highlights and summarizes the two main prongs of the GEMM Family Study: (I) the potential academic and scientific scope of GEMM’s research design; and (II) the implications of the data obtained from the first 16 female healthy volunteers considered as an example of the methodology proposal/development of GEMM’s research design as we anticipate to obtain in our final database. 

I (a). The GEMM Family study’s overarching scientific premise is that inter-individual variation in risk of CVD, T2D and other cardiometabolic diseases is due in part to variation in flexibility and efficiency in disposing of a meal bolus, and this variation may be more readily observed postprandially than at fasting. Our approach includes both a novel, individualized meal challenge (a healthy combination of carbohydrates, protein, and fat calibrated to each subject’s energy requirement) and comprehensive measurement of OMICS data—individualized profiling of gene action in response to the meal. Such a focus on the genetic response following the consumption of a nutritionally defined meal at the level of the specific tissues involved (i.e., fat and muscle), will produce new insights into the genetic architecture of individual variation in metabolism of carbohydrates, fats and proteins, and how this variation in response relates to risk for a variety of chronic diseases including obesity, diabetes and heart disease. 

I (b). GEMM focuses on Mexican nationals. However, genetic epidemiology has shown that Mexicans share with Mexican Americans an elevated prevalence of CVD risk factors [36], suggesting shared genetic factors. As mentioned earlier, Mexico, as the source population, is likely to retain more of the allelic diversity obtained through the admixture from the Conquest, when European and Native Americans began interbreeding. Studying the genetics of CVD risk factors in Mexican nationals could have a strong impact on future public health policies for US-born Mexican Americans or individuals of Mexican origin living in the USA [37]. 

I (c). The GEMM protocol includes an innovative mixed meal challenge [38] containing a well-defined macronutrient composition based on recommended daily values (Ensure Plus^®^) [39], dosed at 30% of each subject’s daily resting energy expenditure allowing a much greater opportunity to screen for early postprandial detection of an adverse metabolic response. It has been documented that risk factors for adverse cardiovascular events can be detected in the pre-diabetic insulin-resistant subject based upon the metabolic response to a meal challenge even in the absence of altered fasting parameters. The superiority of a mixed meal versus the oral glucose tolerance test, related to cardiac dysfunction, has been proposed to relate to the postprandial hypertriglyceridemia which only occurred using the mixed meal [40]. 

I (d). GEMM interrogates the genomic and physiologic basis of postprandial metabolism by measuring dynamic phenotypes. Our study is designed to unravel metabolic responses both at the molecular and physiological level, characterizing a response to a nutritional challenge across a time course and in more than one tissue, which appears to be the better tool to reveal metabolic disturbances, compared to single-point measurements at the static fasting state. Our innovative postprandial study design measures biochemical trajectories that differ from static measurements in the same way motion pictures differ from snapshots: the dimension of time is included. Our approaches for measuring molecular, biochemical, metabolic and clinical dynamics are therefore fundamentally different from the conventional approach of measuring static concentrations [41].

I (e). GEMM uses combined multi-OMIC, multi-tissue data to address hypotheses about variable response to feeding. By repeatedly measuring both biochemical and intermediate molecular markers in the circulation across a time course, we are able to observe individual differences in macronutrient uptake and disposal in unprecedented detail. Similarly, by obtaining high-dimensional transcriptomic, proteomic, and metabolomic measures from skeletal muscle and adipose tissue biopsies from the same individuals at fasting and 3 h after the meal, we will acquire integrated profiles of gene action in response to feeding in a key tissue for macronutrient homeostasis. 

II (a). Regarding our proposal/development data, we reiterate that the reason to present a small number of subjects (*n* = 16) is solely to illustrate the development purposes of the methodology. The changes described in the preliminary results confirm prior studies, including the higher levels of leptin and ghrelin, the increase in the inflammatory/cardiovascular risk marker, CRP, and the presence of insulin resistance in the subjects with higher BMI levels (Table 1). The postprandial leptin, insulin levels and gastrointestinal satiety signals involved in neurohormonal regulation of energetic homeostasis (GLP-1, PYY), the fasting inflammatory biomarkers (TNFα, hs-CRP, PAI-1, IL-6), and the biochemistry markers of lipid-lipoprotein metabolism (NEFA, triglycerides, C-HDL), clearly showed a trend to be elevated in our group with higher BMI values, except for postprandial ghrelin lower levels (Table 1 and Table 2). A low C-HDL, and high levels of NEFA, triglycerides, IL-6, TNF-α and CRP have been previously implicated in the development of insulin resistance [42]. 

II (b). Concordantly, our subjects with a h.BMI were less insulin sensitive than the group with l.BMI. by two independent criteria HOMA-IR [33] and the Matsuda Index [34]. The latter is considered a dynamic measure for whole body insulin sensitivity. 2.5 or less of Matsuda Index have been used to find subjects with insulin resistance [43]. The Matsuda Index in our preliminary data shows a trend of a lower Index in the group with h.BMI. The HOMA-IR derives from measurements of fasting plasma glucose and insulin concentrations primarily reflecting hepatic insulin resistance [44]. The best cutoff of HOMA-IR for identifying Americans of Mexican descent with insulin resistance are reference values <2.60 as the normal range, HOMA-IR 2.60–3.80 as ‘borderline high’ without labeling these individuals as having insulin resistance, and HOMA-IR >3.80 as ‘high’ having clear correlates of insulin resistance [45]. Our group with h.BMI reported a HOMA-IR of 3.68 (Table 2). 

II (c). Table 3 shows our preliminary results on postprandial targeted amino acid signatures. Recent large-scale metabolite profiling studies have highlighted alterations in essential amino acid metabolism that mark the obese, insulin-resistant phenotype [46]. However, most studies that have examined blood amino acid patterns in obesity and T2DM have been conducted in the overnight- to extended-fasting state. There is no evidence that obesity or insulin resistance alters renal processing of blood amino acids, indicating that the observed fasting blood amino acid patterns are not likely a direct reflection of dietary-derived amino acids or differences in urinary excretion of these metabolites. Therefore, it is fair to suggest that all assumptions such as the ones from the results of fasting branched-chain amino acids (BCAA) patterns and insulin resistance should be taken with caution, unless the postprandial state is included as a means of differential comparison with fasting [47]. 

II (d). For our small pilot study of muscle gene expression (Figure 3) we found 32 differentially expressed transcripts in muscle (*p* < 0.05). 15 of those transcripts were upregulated and 7 were downregulated after the mixed meal. Two genes were significantly expressed (*p* < 10^−5^): *PDK4* and *TXNIP*. *PDK4* is abundant in pancreatic islets and in skeletal muscles that have high glucose utilization and fatty acid oxidation rates [48]. It has been recently reported that the gene expression of thioredoxin-interacting protein (*TXNIP*) in the skeletal muscle decreased with caloric restriction and the degree of *TXNIP* downregulation was associated with the rate of glucose disposal during clamp measurements [49].

In summary, GEMM combines integrated, multi-OMIC profiles with novel postprandial intermediate molecular biomarkers to identify biochemical pathways and potential regulatory networks, thereby identifying some of the earliest markers of metabolic dysregulation and CVRMO that may indicate future T2D and CVD. These preliminary results are firm steps which would help define the range of variation in metabolic flexibility, and therefore early risk of cardiometabolic disease, in nominally healthy individuals. The GEMM study should pave the way for the identification of novel biomarkers of cardiometabolic risk which will have a positive impact on public health initiatives for dealing with these serious conditions, both in Mexico and, possibly, in the rapidly-growing Mexican-American community in the US. 

## Figures and Tables

**Table 1:** Fasting and postprandial phenotypes related to body composition, adipocyte biology, incretins, hunger and satiety, and immune system activity.

	Lower BMI Values (*n* = 8)	Higher BMI Values (*n* = 8)
	24 ± 1.6	32.1 ± 5.7
**Anthropometric and Clinical Measurements**		
Age	34.1 ± 14.4	41.2 ± 11.0
Height (cm)	152.4 ± 6.0	151.9 ± 3.6
Weight (kg)	56.1 ± 7.9	74.1 ± 14.0
Waist Circumference (cm)	76.6 ± 4.5	95.3 ± 11.4
Fat Total (%)	39.1 ± 4.5	46.5 ± 4.7
Fat Mass Total (kg)	20.9 ± 5.0	33.4 ± 8.3
Muscle Mass Total (kg)	31.9 ± 3.2	37.8 ± 6.3
Avg. Systolic Pressure (mmHg)	107.7 ± 7.8	109.6 ± 12.5
Avg. Diastolic Pressure (mmHg)	67.5 ± 3.9	70.8 ± 7.7
**Adipose Tissue Hormones**		
Fasting Adiponectin (µg/mL)	19.9 ± 13.2	22.6 ± 19.6
Fasting Leptin (ng/mL)	8.6 ± 5.6	18.6 ± 9.3
Postprandial Leptin (30 min)	7.9 ± 5.1	18.0 ± 9.9
Postprandial Leptin (60 min)	8.1 ± 5.0	17.8 ± 9.4
Postprandial Leptin (120 min)	8.5 ± 5.9	16.6 ± 9.1
Postprandial Leptin (180 min)	9.3 ± 6.1	16.7 ± 8.1
Postprandial Leptin (300 min)	9.4 ± 5.9	16.4 ± 8.2
**Gastrointestinal Satiety Signals**		
Fasting GLP1	121.9 ± 56.8	136.3 ± 45.5
Postprandial GLP1 30 min	147.9 ± 48.8	189.5 ± 63.1
Postprandial GLP1 60 min	123.0 ±47.6	151.1 ± 58.2
Postprandial GLP1 120 min	130.4 ± 53.1	143.8 ± 64.2
Postprandial GLP1 180 min	120.4 ± 49.1	138.8 ± 51.6
Postprandial GLP1 300 min	123.1 ± 61.8	145.0 ± 64.6
Fasting PYY	54.2 ± 46.5	35.1± 51.2
Postprandial PYY 30 min	70.6 ± 24.8	121.2 ± 74.8
Postprandial PYY 60 min	83.8 ± 65.3	94.3 ± 52.8
Postprandial PYY 120 min	75.7 ± 52.1	79.7 ± 51.8
Postprandial PYY 180 min	82.1 ± 56.3	86.8 ± 63.7
Postprandial PYY 300 min	75.8 ± 44.8	76.1 ± 66.5
Fasting Ghrelin	333.5 ± 59.1	233.1 ± 137.2
Postprandial Ghrelin 30 min	274.1 ± 50.2	192.2 ± 127.2
Postprandial Ghrelin 60 min	220.9 ± 41.2	166.1 ± 116.6
Postprandial Ghrelin 120 min	206.7 ± 44.6	155.6 ± 106.1
Postprandial Ghrelin 180 min	211 ± 66.4	152.9 ± 108.9
Postprandial Ghrelin 300 min	249.6 ± 76.4	157.2 ± 124.8
**Adipocytokines and Inflammatory Biomarkers**		
TNFα	1.4 ± 1.1	1.8 ± 0.7
hs-CPR	0.09 ± 0.07	0.42 ± 0.30
PAI-1	43,527.9 ± 38,563.4	66,901.4 ± 56,005.3
IL-6	1.9 ± 1.3	2.1 ± 1.6

BMI: body mass index; avg: average.

**Table 2:** Fasting and postprandial phenotypes related to the insulin-glucose axis, lipid-lipoprotein metabolism and insulin-mediated glucose disposal.

	Lower BMI Values (*n* = 8)	Higher BMI Values (*n* = 8)
	24 ± 1.6	32.1 ± 5.7
**Insulin-Glucose Axis**		
Fasting Glucose (mg/dL)	84.3 ± 4.9	87.9 ± 7.0
Postprandial Glucose (30 min) (mg/dL)	116.5 ± 9.57	107.8 ± 11.4
Postprandial Glucose (60 min) (mg/dL)	119.25 ± 27.09	115.62 ± 13.83
Postprandial Glucose (120 min) (mg/dL)	114.5 ± 17.07	115.62 ± 19.15
Postprandial Glucose (180 min) (mg/dL)	101.25 ± 16.42	99.37 ± 9.83
Postprandial Glucose (300 min) (mg/dL)	93.62 ± 14.16	92.25 ± 4.65
Fasting Insulin (uIU/mL)	11.04 ± 10.01	16.64 ± 10.41
Postprandial Insulin (30 min) (uIU/mL)	63.81 ± 29.78	78.29 ± 42.78
Postprandial Insulin (60 min) (uIU/mL)	72.53 ± 42.82	84.03 ± 60.51
Postprandial Insulin (120 min) (uIU/mL)	51.09 ± 22.65	54.27 ± 36.19
Postprandial Insulin (180 min) (uIU/mL)	31.46 ± 12.45	41.85 ± 25.79
Postprandial Insulin (300 min) (uIU/mL)	16.94 ± 14.40	27.65 ± 18.52
HOMA-IR	2.31 ± 2.17	3.68 ± 2.34
Matsuda Index	6.66 ± 4.05	4.89 ± 3.59
**Lipid-Lipoprotein Metabolism**		
Fasting NEFA (mEq/L)	0.7 ± 0.1	0.7 ± 0.1
Postprandial NEFA (30 min) (mEq/L)	0.5 ± 0.2	0.7 ± 0.1
Postprandial NEFA (60 min) (mEq/L)	0.2 ± 0.1	0.4 ± 0.2
Postprandial NEFA (120 min) (mEq/L)	0.1 ± 0.1	0.2 ± 0.1
Postprandial NEFA (180 min) (mEq/L)	0.1 ± 0.1	0.3 ± 0.2
Postprandial NEFA (300 min) (mEq/L)	0.5 ± 0.3	0.5 ± 0.2
Fasting Triglyceride (mg/dL)	89.9 ± 27.2	161.7 ± 47.0
Postprandial Triglyceride (30 min) (mg/dL)	84.3 ± 27.0	157.6 ± 33.6
Postprandial Triglyceride (60 min) (mg/dL)	90.6 ± 27.3	165.1 ± 41.1
Postprandial Triglyceride (120 min) (mg/dL)	92.4 ± 31.2	186.8 ± 55.4
Postprandial Triglyceride (180 min) (mg/dL)	103.0 ± 39.1	209.1 ± 75.3
Postprandial Triglyceride (300 min) (mg/dL)	108.1 ± 44.0	204.8 ± 61.2
Fasting HDL-Col	58.5 ± 13.6	48.9 ± 6.4
Postprandial HDL-Col (180 min)	55.0 ± 14.3	46.8 ± 6.3
Postprandial HDL-Col (300 min)	55.6 ± 15.1	46.3 ± 6.6

NEFA: non-esterified free fatty acids; HDL-C: High-density lipoprotein cholesterol.

**Table 3:** Fasting and postprandial phenotypes related to metabolomics, amino acid signatures and acylcarnitine.

	Lower BMI Values (*n* = 8)	Higher BMI Values (*n* = 8)
	24 ± 1.6	32.1 ± 5.7
**Metabolomics Profiling**		
Targeted Aminoacid Signature		
Branched-chain (BCCA) amino acids		
Isoleucine 0 min	42.5 ± 7.2	41.8 ± 5.0
Isoleucine 30 min	73.3 ± 14.5	51.6 ± 11.28
Isoleucine 180 min	63.1 ± 10.8	50.1 ± 8.5
Isoleucine 300 min	55.5 ± 6.6	50.6 ± 9.7
Leucine 0 min	77.8 ± 10.2	80.8 ± 14.7
Leucine 30 min	125.3 ± 19.8	95.6 ± 21.1
Leucine 180 min	99.8 ± 13.6	85.8 ± 15.6
Leucine 300 min	88.1 ± 9.7	81.02 ± 12.9
Valine 0 min	141.9 ± 24.7	153.1 ± 38.9
Valine 30 min	183.9 ± 34.9	167.1 ± 47.8
Valine 180 min	171.4 ± 22.2	159.4 ± 36.6
Valine 300 min	162.4 ± 11.2	159.5 ± 32.3
Phenylalanine 0 min	43.05 ± 3.13	53. 7 ± 8.1
Phenylalanine 30 min	57.3 ± 5.7	59.6 ± 8.8
Phenylalanine 180 min	53.9 ± 4.9	56.9 ± 7.8
Phenylalanine 300 min	48.2 ± 5.01	55.3 ± 7.5
Aspartic acid 0 min	3.9 ± 2.1	6.1 ± 1.6
Aspartic acid 30 min	2.7 ± 1.7	3.7 ± 2.2
Aspartic acid 180 min	2.3 ± 1.5	2.5 ± 2
Aspartic acid 300 min	3.05 ± 1.2	4.6 ± 2.5
**Targeted Acylcarnitine Signature**		
Acylcarnitine (14:0) 0 min	0.2 ± 0.07	0.2 ± 0.06
Acylcarnitine (14:0) 30 min	0.2 ± 0.6	0.2 ± 0.06
Acylcarnitine (14:0) 180 min	0.07 ± 0.02	0.1 ± 0.07
Acylcarnitine (14:0) 300 min	0.09 ± 0.06	0.1 ± 0.05
Acylcarnitine (14:1) 0 min	0.7 ± 0.5	0.1 ± 0.2
Acylcarnitine (14:1) 30 min	0.7 ± 0.5	0.1 ± 0.1
Acylcarnitine (14:1) 180 min	0.2 ± 0.2	0.04 ± 0.04
Acylcarnitine (14:1) 300 min	0.4 ± 0.4	0.03 ± 0.02
Acylcarnitine (14:2) 0 min	0.6 ± 0.3	0.2 ± 0.05
Acylcarnitine (14:2) 30 min	0.4 ± 0.2	0.2 ± 0.06
Acylcarnitine (14:2) 180 min	0.2 ± 0.08	0.2 ± 0.09
Acylcarnitine (14:2) 300 min	0.3 ± 0.3	0.2 ± 0.02
Acylcarnitine (14:3) 0 min	0.06 ± 0.03	0.03 ± 0.005
Acylcarnitine (14:3) 30 min	0.05 ± 0.02	0.02 ± 0.003
Acylcarnitine (14:3) 180 min	0.03 ± 0.01	0.02 ± 0.01
Acylcarnitine (14:3) 300 min	0.05 ± 0.04	0.02 ± 0.005
Acylcarnitine (16:0) 0 min	1.6 ± 0.2	1.5 ± 0.3
Acylcarnitine (16:0) 30 min	1.4 ± 0.2	1.5 ± 0.3
Acylcarnitine (16:0) 180 min	0.8 ± 0.2	1.2 ± 0.4
Acylcarnitine (16:0) 300 min	0.8 ± 0.1	1.1 ± 0.3

**Table 4:** Gene expression nominally up- or down-regulated at 3 h postprandium relative to fasting levels in nine female subjects (*t*-test, *p* < 0.05).

Probe	Accession	Gene Symbol	*p*-Value	Direction of Change
ILMN_1684982	NM_002612.3	*PDK4*	0.00000106	up
ILMN_1697448	NM_006472.2	*TXNIP*	0.0000279	up
ILMN_1791728	NM_052901.2	*SLC25A25*	0.000232157	down
ILMN_1663092	NM_006079.3	*CITED2*	0.000454775	up
ILMN_1691846	NM_015714.2	*G0S2*	0.000530702	down
ILMN_1661519	NM_014702.3	*KIAA0408*	0.000559434	up
ILMN_1907042	AK123264	*C1orf132*	0.000903385	up
ILMN_1794017	NM_013376.3	*SERTAD1*	0.00102915	down
ILMN_1728699	NM_194285.2	*SPTY2D1*	0.001347097	down
ILMN_1874689	AB074162	*MIR181A2HG*	0.001811776	down
ILMN_1797031	NM_024610.4	*HSPBAP1*	0.002114313	up
ILMN_1860963	BM715829	transcribed locus	0.00277532	down
ILMN_1710284	NM_005524.2	*HES1*	0.002894267	down
ILMN_1839665	BQ186372	transcribed locus	0.002993048	up
ILMN_1908530	AW003529	*miR-205*	0.003042844	down
ILMN_1776483	GDS5231	no annotation	0.003499501	up
ILMN_1689212	NM_001010892.1	*RSHL3*	0.004134486	down
ILMN_2186061	NM_004566.2	*PFKFB3*	0.004236655	up
ILMN_1870041	AJ227862	partial mRNA	0.004322057	up
ILMN_1771618	NM_173671.1	*FLJ37396*	0.004436233	down
ILMN_3266471	GDS5431	no annotation	0.005104923	down
ILMN_2077680	NM_152353.1	*CLDND2*	0.005149724	up
ILMN_1764873	NM_001419.2	*ELAVL1*	0.005480716	down
ILMN_1756006	NM_015104.1	*ATG2A*	0.005654292	up
ILMN_3262439	GDS5167	no annotation	0.006327375	down
ILMN_1732750	NM_016565.2	*CHCHD8*	0.006543098	down
ILMN_1704022	NM_207316.1	*TMEM207*	0.006752015	down
ILMN_2044927	NM_006913.2	*RNF5*	0.007227478	down
ILMN_1741392	NM_000387.3	*SLC25A20*	0.007466004	up
ILMN_1786242	GDS3855	no annotation	0.008237115	up
ILMN_1836309	GDS3531	no annotation	0.008873484	down
ILMN_3245678	NC_000001.11	*RNU1-1*	0.009334803	up
